# Treatment of people diagnosed with chronic hepatitis C virus infection

**DOI:** 10.2471/BLT.18.219295

**Published:** 2018-08-01

**Authors:** Marc Bulterys, Saeed Sadiq Hamid

**Affiliations:** a Department of HIV and Global Hepatitis Programme, World Health Organization, avenue Appia 20, 1211 Geneva 27, Switzerland.; b The Aga Khan University and Hospital, Karachi, Pakistan

In 2015, 71 million people worldwide were living with one of the six major genotypes of hepatitis C virus (HCV) and 399 000 people died from the infection.^1^ The 2016 World Health Organization (WHO) guidelines for the screening, care and treatment of people with chronic hepatitis C virus infection^2^ focused on two points. First, WHO recommended considering all infected adults, that is, 18 years of age or older, for treatment, but prioritizing those at highest risk of death or disability or those at high risk of transmitting the virus. Second, WHO recommended treating patients with direct-acting antivirals, using different regimens depending on hepatitis C virus genotype.

Since then, three key developments prompted changes in terms of when to treat and what treatment to use. First, safe and highly effective direct acting antiviral regimens that improve the balance of benefit-to-harm of treating people with little or no hepatic fibrosis became the norm for the treatment of all patients. Second, several new, pan-genotypic direct acting antiviral medicines became available, reducing the need for genotyping to guide treatment decisions. Third, the price of direct acting antivirals has fallen considerably, which enabled a more rapid roll-out of treatment in low- and middle-income countries.^3^

New WHO guidelines were launched on World Hepatitis Day^4^ in July 2018. These guidelines are intended for public health officials to use as the basis for developing national hepatitis policies, plans and treatment guidelines.

WHO formulated population, intervention, comparator and outcome (PICO) questions that addressed when to start treatment and what treatment to use. These questions were considered for adults 18 years of age or older, adolescents 12–17 years of age and children younger than 12 years. WHO commissioned systematic reviews, convened a guidelines development group in September 2017, drafted the guidelines and submitted them to peer review and ultimately for clearance by the WHO Guidelines Review Committee.

The 2018 guidelines offer new recommendations in the treatment of hepatitis C virus. First, WHO recommends offering treatment to all adolescents and adults diagnosed with chronic hepatitis C virus infection, with the exception of pregnant women, irrespective of disease stage. Second, the guidelines recommend the use of pangenotypic direct acting antivirals in adults. This includes those who have never been treated with direct-acting antivirals and those who previously received interferon and/or ribavirin. These regimens and the duration of treatment depend on the presence or absence of cirrhosis. In adolescents, WHO recommends direct-acting antiviral-based regimens that are still genotype-dependent. Treatment in adolescents still requires non pangenotypic regimens, and ribavirin for treatment of genotype 2 and 3. Third, in children diagnosed with chronic hepatitis C virus infection, WHO recommends deferring treatment until children reach 12 years of age. None of the recommended pangenotypic direct acting antiviral regimens for adults are yet approved for use in adolescents and children, but approval is anticipated in 2019.

The implications of these new WHO recommendations for public health are threefold. First, treating all persons infected will allow faster progress towards hepatitis C virus elimination as a major public health threat by 2030.^5^ Second, the use of pangenotypic regimens removes the need for expensive genotyping before treatment initiation. Third, treatment of adolescents means that more people could become adults free of hepatitis C virus infection. Further efforts to increase testing, especially in populations most affected by hepatitis C, will contribute to identifying those infected and in need of treatment.^6^ Five population groups (people who inject drugs, people in prisons or other closed settings, men who have sex with men, sex workers and indigenous populations) require specific public health approaches because of stigma and discrimination, vulnerability and difficulties in accessing services. Treating all persons diagnosed with hepatitis C virus infection using pangenotypic direct acting antiviral regimens will simplify care and facilitate achieving viral hepatitis elimination as established in the *Global health sector strategy on viral hepatitis*, approved in 2016.^5^
